# Machine learning-based multimodal MRI texture analysis for assessing renal function and fibrosis in diabetic nephropathy: a retrospective study

**DOI:** 10.3389/fendo.2023.1050078

**Published:** 2023-04-17

**Authors:** Wenbo Chen, Lu Zhang, Guanhui Cai, Bin Zhang, Zhouyang Lian, Jing Li, Wenjian Wang, Yuxian Zhang, Xiaokai Mo

**Affiliations:** ^1^ Department of Radiology, The First Affiliated Hospital of Jinan University, Guangzhou, Guangdong, China; ^2^ Department of Radiology, Huizhou Central People’s Hospital, Huizhou, Guangdong, China; ^3^ Department of Radiology, Guandong Academy of Medical Sciences/Guangdong Provincial People’s Hospital, Guangzhou, Guangdong, China; ^4^ Division of Nephrology, Guangdong Academy of Medical Sciences/Guangdong Provincial People’s Hospital, Guangzhou, Guangdong, China; ^5^ School of Medicine, South China University of Technology, Guangzhou, Guangdong, China; ^6^ Department of Nuclear Medicine, ZhuJiang Hospital of Southern Medical University, Guangzhou, Guangdong, China

**Keywords:** texture analysis, multimodal MRI (mMRI), machine learning, diabetic nephropathy (DN), functional MRI (fMRI)

## Abstract

**Introduction:**

Diabetic nephropathy (DN) has become a major public health burden in China. A more stable method is needed to reflect the different stages of renal function impairment. We aimed to determine the possible practicability of machine learning (ML)-based multimodal MRI texture analysis (mMRI-TA) for assessing renal function in DN.

**Methods:**

For this retrospective study, 70 patients (between 1 January 2013 and 1 January 2020) were included and randomly assigned to the training cohort (*n*1 = 49) and the testing cohort (*n*2 = 21). According to the estimated glomerular filtration rate (eGFR), patients were assigned into the normal renal function (normal-RF) group, the non-severe renal function impairment (non-sRI) group, and the severe renal function impairment (sRI) group. Based on the largest coronal image of T2WI, the speeded up robust features (SURF) algorithm was used for texture feature extraction. Analysis of variance (ANOVA) and relief and recursive feature elimination (RFE) were applied to select the important features and then support vector machine (SVM), logistic regression (LR), and random forest (RF) algorithms were used for the model construction. The values of area under the curve (AUC) on receiver operating characteristic (ROC) curve analysis were used to assess their performance. The robust T2WI model was selected to construct a multimodal MRI model by combining the measured BOLD (blood oxygenation level-dependent) and diffusion-weighted imaging (DWI) values.

**Results:**

The mMRI-TA model achieved robust and excellent performance in classifying the sRI group, non-sRI group, and normal-RF group, with an AUC of 0.978 (95% confidence interval [CI]: 0.963, 0.993), 0.852 (95% CI: 0.798, 0.902), and 0.972 (95% CI: 0.995, 1.000), respectively, in the training cohort and 0.961 (95% CI: 0.853, 1.000), 0.809 (95% CI: 0.600, 0.980), and 0.850 (95% CI: 0.638, 0.988), respectively, in the testing cohort.

**Discussion:**

The model built from multimodal MRI on DN outperformed other models in assessing renal function and fibrosis. Compared to the single T2WI sequence, mMRI-TA can improve the performance in assessing renal function.

## 1 Introduction

Diabetic nephropathy (DN) is a serious complication of diabetes that results in renal failure. In recent decades, DN has become a major public health crisis and economic burden for dialysis to keep patients alive in China. The outcome of DN is chronic progressive kidney damage and renal fibrosis. At present, the assessment of renal function on DN mainly relies on the estimated glomerular filtration rate (eGFR). However, serum creatinine can be easily affected by medications or metabolism (1). Moreover, the eGFR increased at the early stage of DN for compensation, and it was easily neglected clinically (2). However, renal function decline and subsequent renal fibrosis in DN are irreversible. The eGFR may be lagged for the exact renal dysfunction in DN. Therefore, a more stable method is needed to reflect the different stages of renal function impairment.

With the development of imaging technology, functional MRI (fMRI), including diffusion-weighted imaging (DWI) and blood oxygenation level–dependent MRI (BOLD), has been used to detect early renal function impairment and assess renal fibrosis (3, 4). BOLD uses endogenous deoxyhemoglobin in the vessel as a contrast to detect MRI signal changes, which can reflect renal hypoxia (5). The higher R2* values suggest more hypoxia in the tissue. Meanwhile, DWI provides information about tissue microarchitecture by evaluating the Brownian motion of water molecules (6–8); tissues with restricted extracellular water content exhibit higher signal intensity in DWI and lower apparent diffusion coefficient (ADC) values (9). The lower ADCs were correlated with renal fibrosis in DN progression. Functional MRIs are stable and non-invasive methods to reflect the pathophysiology of DN, including renal hypoxia in early renal dysfunction and renal fibrosis as disease progression (10). Therefore, fMRIs are good alternative methods for renal function assessment.

Artificial intelligence (AI) is an emerging technology and a current research hotspot that uses software technologies to collect more medical information than the human eyes can. AI has played an important role in medical diagnosis and treatment; it might reduce the number of medical errors and misdiagnoses and possibly increase the quality of patient management (11). Researchers have used AI techniques for detecting lung nodules in patients with complex lung disease (12), segmentation in prostate radiation therapy (13), diagnosis of metastatic lymph nodes in patients with papillary thyroid cancer (14), predicting response of radiotherapy in patients with locally advanced rectal cancer (15), and so on.

Machine learning studies the theory of pattern recognition and computational learning in AI, which focuses on studying huge amounts of data with multiple variables (16). Imaging textures can be extracted to reflect tissue heterogeneity (17–19). Imaging textures have been used in predicting the renal function of the transplanted kidney on T2WI, detecting early renal damage by DTI, and classifying the renal function status on several MRI sequences (20–22). Previous studies have suggested that texture features might be feasible in the assessment of renal dysfunction on functional MRI (22). Generally, the conventional imaging sequence is not well investigated on renal MRI. On the other hand, they did not establish a machine learning-based model to integrate significant imaging textures (20–22). Therefore, we would like to incorporate the conventional T2WI and fMRI to establish a machine learning-based multimodal MRI model to evaluate renal function in DN.

In our study, we aimed to explore the value of machine learning-based texture analysis based on T2WI and integrate the measured BOLD and DWI values to assess renal function in DN.

## 2 Materials and methods

### 2.1 Study design

This retrospective study was approved by the local institutional review board of Guangdong Provincial People’s Hospital. Written informed consent was obtained from each participant. A total of 70 patients (between 1 January 2013 and 1 January 2020) diagnosed as DN clinically were eligible for enrollment, and they had undergone mid-abdominal MRI examination. Patients with giant kidney tumor, polycystic kidney, or contraindications of MRI examination (i.e., body habitus incompatible with MRI equipment, presence of ferrous metallic implants, or claustrophobia) were excluded. Serum creatinine was obtained within 7 days of MRI examination and eGFR was calculated by CKD-EPI (chronic kidney disease, Epidemiology Collaboration equations). According to the values of eGFR, patients were divided into normal renal function (normal-RF) with CKD1 (eGFR ≥ 90 ml/min/1.73 m^2^), non-severe renal function impairment (non-sRI) with CKD2 and CKD3 (30 ml/min/1.73 m^2^ ≥ eGFR < 90 ml/min/1.73 m^2^), and severe renal function impairment (sRI) with CKD4 and CKD5 (eGFR < 30 ml/min/1.73 m^2^). They were randomly assigned to the training cohort (*n*1 = 49) and testing cohort (*n*2 = 21).

### 2.2 MRI protocol

All patients underwent the examination on the 3.0-T whole-body system (Signa EXCITE HD, GE Healthcare, Milwaukee, WI, USA). The protocol of coronal T2WI was as follows: TR/TE = 1,300/80 (ms), field of view = 36.0 (mm^2^), matrix = 512 × 512, slice thickness = 3.0 mm, flip angle = 90°, and bandwidth = 50.0. The protocol of BOLD was as follows: TR/TE = 112/3–30 (ms), field of view = 36.0 (mm^2^), matrix = 512 × 512, slice thickness = 3.0 mm, flip angle = 60°, and bandwidth = 50.0. The protocol of DWI was as follows: TR/TE = 2,600/80, field of view = 36.0 (mm^2^), matrix = 512 × 512, slice thickness = 3.0 mm, and bandwidth = 75.0.

### 2.3 MRI imaging analysis and model construction

The kidneys were selected and segmented by ITK-SNAP software (https://itk.org/). The largest coronal slice crossing the renal hilum of the renal parenchyma on T2WI was outlined. The speeded up robust features (SURF) algorithm was used for texture feature extraction. The feature selection techniques, namely, ANOVA and relief and recursive feature elimination (RFE), were applied to select the important features. Both imaging features and clinical characteristics including age, gender, and body mass index (BMI) were entered into the support vector machine (SVM), logistic regression (LR), and random forest (RF) algorithms to build a robust T2WI-based model for renal function evaluation. The robust T2WI-based model with best performance was selected to build the multimodal MRI model.

**Figure 1:**
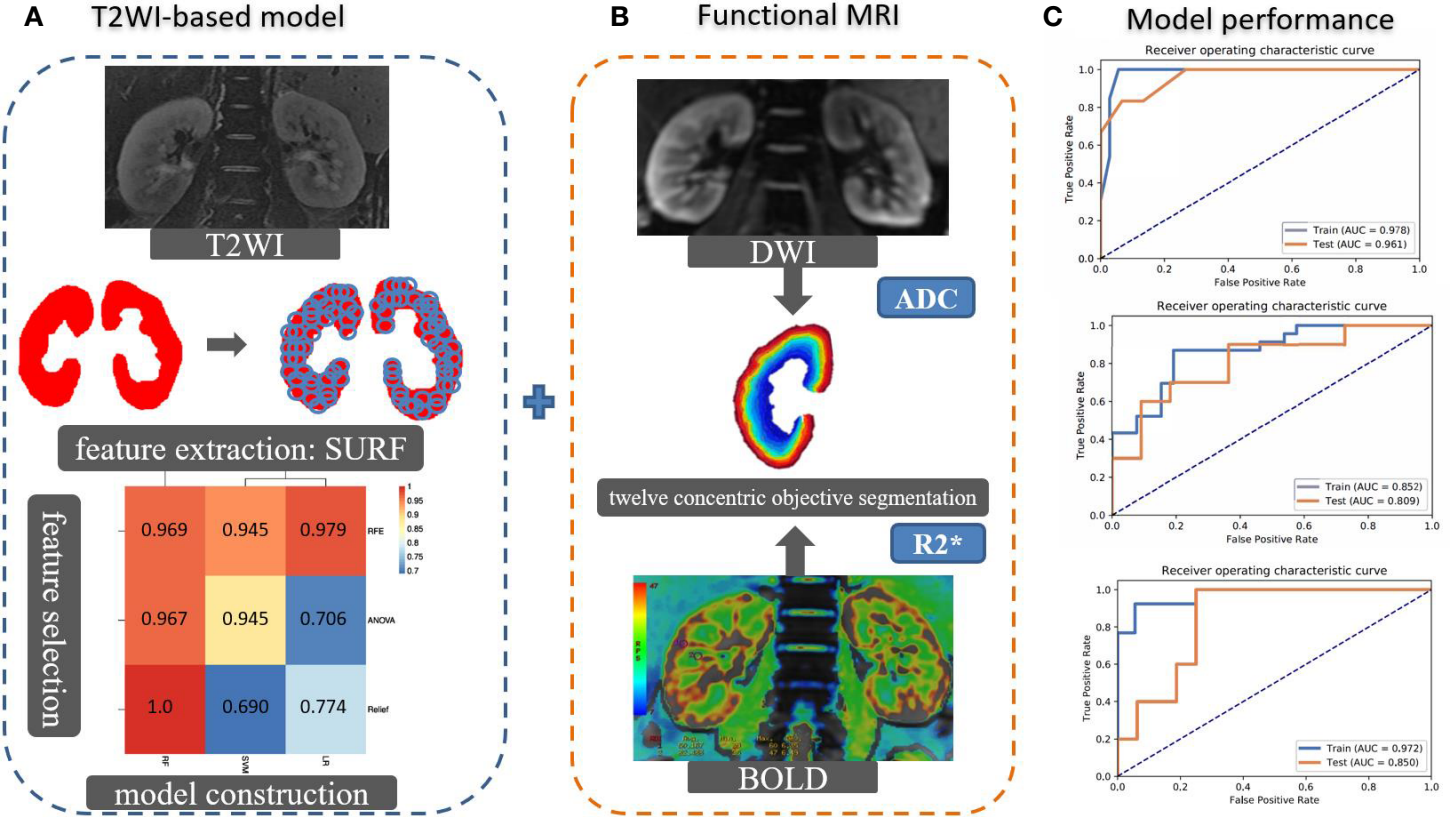
The flowchart of building models to classify the renal function status. **(A)** The SURF was used for texture feature extraction in the largest coronal slice crossing the renal hilum on T2WI sequence. Several machine learning algorithms were used to select important features and construct models. Imaging features and clinical characteristics were incorporated to build the T2WI-based model. **(B)** R2* and ADC values were measured by 12 concentric objective segmentations on the right kidney from BOLD and DWI, respectively. The mMRI-TA model was constructed by combining the T2WI-based model, R2*, and ADC values. **(C)** The ROCs were used to demonstrate the model performance. SURF: speeded up robust features.

The R2* and ADC values were measured by 12 concentric objective segmentations on the right kidney on BOLD and DWI, respectively (23). Then, we incorporated the T2WI-based model, R2*, and ADCs by the machine learning algorithm to establish a multimodal MRI model for stratifying the renal function. The performance of constructed models was evaluated by area under the curve (AUC) on receiver operating characteristic (ROC) curve analysis on classifying the sRI, non-sRI, and normal renal function groups. The flowchart of building models to classify the renal function status is shown in **Figure 1**.

### 2.4 Statistical analysis

The clinical characteristics were compared in the training and testing cohorts, using independent samples *t*-test, *χ* ^2^ test, and Wilcoxon rank sum test when appropriate. AUCs were used to evaluate the performance of models. Two-sided *p* < 0.05 was considered significant. All statistical analyses were implemented in 2018 Python 3.6.5 (https://www.python.org/). The “NumPy” package was used to standardize the intensity range of T2WI. “OpenCV” was applied for image masking, kidney segmentation, and feature extraction. “Scikit-learn” package was used for feature selection (ANOVA, RFE, and Relief) and model construction (SVM, RF, and LR). The ROC curves were plotted by the “matplotlib” package.

### 2.5 Patient and public involvement

No patients were actively involved in setting the research question and outcome measures, nor were they involved in the design of the study. Patients were not involved in the interpretation or write-up of the results, nor are there plans for the results to be disseminated to the patient community affected by this research.

## 3 Results

### 3.1 Patient characteristics

Patients were randomly assigned into a training cohort (*n*1 = 49) and a testing cohort (*n*2 = 21). Their age, gender, and BMI were compared. No significant difference in sRI, non-sRI, and normal renal function groups was found between the training and testing cohorts (**Table 1**).

**Table 1:** The clinical characteristics of training and testing cohorts.

		Train (*n* = 49)	Test (*n* = 21)	*p*
Groups, *n* (%)	Normal-RF	13 (27%)	5 (24%)	0.967
	Non-sRI	23 (47%)	10 (47%)	
	sRI	13 (26%)	6 (29%)	
Gender, *n* (%)	Female	19 (39%)	7 (33%)	0.666
	Male	30 (61%)	14 (67%)	
Age, mean ( ± SD)		56.7 ± 10.2	58.2 ± 9.2	0.568
BMI, mean ( ± SD)		23.488 ± 3.644	25.241 ± 2.921	0.059

BMI, body mass index.

### 3.2 Performance of the T2WI-based model to identify normal-RF, non-sRI, and sRI

**Figure 2:**
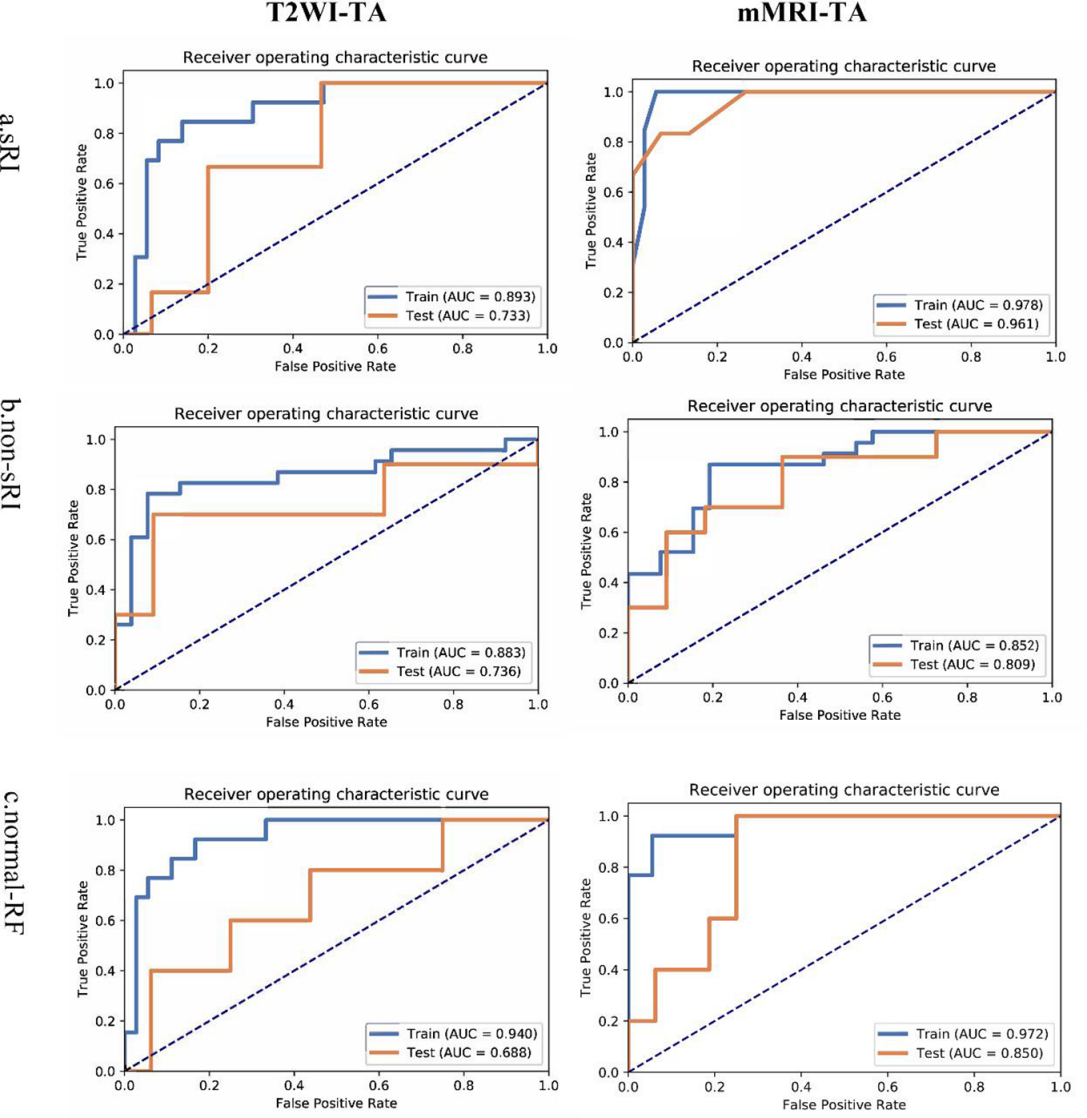
ROCs of T2WI and mMRI-TA models to identify normal-RF, non-sRI, and sRI. normal-RF: normal renal function; non-sRI: non-severe renal function impairment; sRI: severe renal function impairment.

The T2WI-based model showed satisfying performance in identifying non-sRI, sRI, and normal-RF in the training and testing cohorts. ROCs were plotted in **Figure 2**. Among the three feature selection techniques, ANOVA, Relief, and RFE, and the three machine learning classification algorithms, SVM, LR, and RF, we found that the ANOVA and SVM model showed robust performance in classifying sRI, non-sRI, and normal-RF groups (**Table 2**). Using the ANOVA+SVM model in identifying the normal-RF group, the AUC was 0.940 (95% confidence interval [CI], 0.902, 0.969) in the training cohort, while it was 0.688 (95% CI, 0.378, 0.947) in the testing cohort. In identifying the non-sRI group, the AUC was 0.883 (95% CI, 0.830, 0.928) and 0.736 (95% CI, 0.455, 0.963), respectively, in the training and testing cohorts. In identifying the sRI group, the AUC showed 0.893 (95% CI, 0.804, 0.926) in the training cohort and 0.733 (95% CI, 0.513, 0.947) in the testing cohort.

**Table 2:** Performance of the T2WI-based model to identify normal-RF, non-sRI, and sRI in training and testing cohorts.

	Training cohort			Testing cohort		
	SVM	RF	LR	SVM	RF	LR
sRI						
ANOVA	0.893 (0.804, 0.926)	1.000 (1.000, 1.000)	0.887 (0.828, 0.937)	0.733 (0.513, 0.947)	0.844 (0.635, 1.000)	0.733 (0.513, 0.947)
RFE	0.869 (0.804, 0.926)	1.000 (1.000, 1.000)	0.887 (0.828, 0.937)	0.733 (0.513, 0.947)	0.833 (0.612, 0.981)	0.733 (0.513, 0.947)
Relief	0.961 (0.937, 0.981)	1.000 (1.000, 1.000)	0.929 (0.892, 0.959)	0.756 (0.500, 0.971)	0.800 (0.575, 0.978)	0.778 (0.559, 0.962)
Non-sRI						
ANOVA	0.883 (0.830, 0.928)	0.967 (0.942, 0.987)	0.706 (0.632, 0.775)	0.736 (0.455, 0.963)	0.818 (0.592, 1.000)	0.582 (0.338, 0.855)
RFE	0.945 (0.913, 0.972)	0.969 (0.960, 0.993)	0.979 (0.957, 0.994)	0.736 (0.454, 0.967)	0.505 (0.250, 0.765)	0.727 (0.444, 0.967)
Relief	0.690 (0.615, 0.766)	1.000 (1.000, 1.000)	0.774 (0.699, 0.842)	0.489 (0.235, 0.726)	0.532 (0.245, 0.789)	0.612 (0.346, 0.863)
Normal-RF						
ANOVA	0.940 (0.902, 0.969)	1.000 (1.000, 1.000)	0.949 (0.919, 0.975)	0.688 (0.378, 0.947)	0.663 (0.370, 0.914)	0.688 (0.370, 0.947)
RFE	0.992 (0.981, 0.999)	1.000 (1.000, 1.000)	0.991 (0.981, 0.998)	0.775 (0.463, 1.000)	0.682 (0.346, 0.950)	0.800 (0.482, 1.000)
Relief	0.896 (0.837, 0.950)	1.000 (1.000, 1.000)	0.939 (0.902, 0.973)	0.537 (0.167, 0.868)	0.675 (0.414, 0.8824)	0.525 (0.204, 0.926)

normal-RF, normal renal function; non-sRI, non-severe renal function impairment; sRI, severe renal function impairment; SVM, support vector machine; LR, logistic regression; RF, random forest, ANOVA, analysis of variance; RFE, recursive feature elimination.

### 3.3 Performance of the mMRI-TA model to identify normal-RF, non-sRI, and sRI

On the basis of the T2WI-based model, we had incorporated the quantitative values on BOLD and DWI to form the mMRI-TA model. The mMRI-TA model showed excellent performance in identifying normal-RF, non-sRI, and sRI in the training and testing cohorts (**Table 3**), and ROCs are demonstrated in **Figure 2**. In identifying the normal-RF group, the AUC was 0.972 (95% CI, 0.995, 1.000) in the training cohort and 0.850 (95% CI, 0.638, 0.988) in the testing cohort. In identifying the non-sRI group, the AUC was 0.852 (95% CI, 0.798, 0.902) in the training cohort and 0.809 (95% CI, 0.600, 0.980) in the testing cohort. In identifying the sRI group, the AUC was 0.978 (95% CI, 0.963, 0.993) in the training cohort and 0.961 (95% CI, 0.853, 1.000) in the testing cohort.

**Table 3:** Performance of the mMRI-TA model to identify normal-RF, non-sRI, and sRI in training and testing cohorts.

	Training cohort	Testing cohort
**sRI**	0.978 (0.963, 0.993)	0.961 (0.853, 1.000)
**Non-sRI**	0.852 (0.798, 0.902)	0.809 (0.600, 0.980)
**Normal-RF**	0.972 (0.995, 1.000)	0.850 (0.638, 0.988)

normal-RF, normal renal function; non-sRI, non-severe renal function impairment; sRI, severe renal function impairment.

## 4 Discussion

Our study showed that the mMRI-TA model derived from T2WI and measured DWI and BOLD values was able to identify non-sRI, sRI, and normal-RF groups. We have investigated the performance of texture-based models on T2WI and functional MRI on renal function classification in DN.

Texture feature analysis on renal ultrasound and MRI can classify the renal function and the chronic kidney disease progression (24, 25). Several studies had confirmed that the decreased ADCs and increased R2* were detected in patients with renal dysfunction (26–28). The changes in signal intensity on DWI and BOLD were significantly associated with the area of fibrosis and cell density during renal fibrogenesis, and the degree of fibrosis was significantly associated with the status of renal function (28, 29). However, the manual ROIs were variable in previous renal studies, which affect the performance of fMRI (22, 30, 31). It is difficult for the human naked eyes to identify the changes of T2WI in renal fibrosis. Therefore, we used AI technology to detect the changes in signal intensity in patients with renal dysfunction and fibrosis by extracting a great number of MR imaging textures and further texture feature analysis. For renal fibrosis, the glomerulosclerosis and tubulointerstitial fibrosis were present, leading to decreased signal intensity on T2WI. Those subtle changes can be quantified by the imaging textures on T2WI. Therefore, the texture analysis on T2WI yielded good performance in stratifying the renal function status.

For the DWI and BOLD, we measured the values by a semi-automatic method to obtain stable data. Renal fibrosis is probably one of the main causes of decreased ADC values during renal function impairment (32). Reduced renal blood flow and renal fibrosis make a contribution to the progression of renal dysfunction (33, 34). On the other hand, the BOLD sequence is able to reflect hypoxia in the renal microenvironment. The renal medulla contains a great number of mitochondria, which are the most energy-consuming tubular cells. Hypoxia can be detected by BOLD in the medulla earlier with intense activity over the course of renal injury (2). The measured R2* and ADC values can reflect renal hypoxia and fibrosis, which may be an effective supplement to the T2WI-based model. Therefore, the mMRI-TA model constructed by T2WI and measured BOLD and DWI values has improved the performance of the T2WI-based model in assessing renal function.

Different machine learning methods have advantages and disadvantages in different applications. Some studies have compared machine learning methods to assess renal function in chronic kidney disease. Researchers have compared six algorithms, LR, RF, SVM, K nearest neighbors (KNN), Naive Bayes (NB), and feed-forward neural network (FNN), and RF achieved the best performance (35). Some authors used the RFE feature selection method and four classification algorithms (SVM, KNN, DT, and RF), and found that RF outperformed all other algorithms in CKD diagnosis (36). While other researchers detected CKD by combining the information gain-based feature selection technique and adaptive boosting (AdaBoost) classifier, they found that the cost-sensitive AdaBoost trained with the reduced feature set achieved the best classification performance with an accuracy of 99.8% (37). In our study, we have used the SVM, RF, and LR to build models and found that the SVM model is more stable in categorizing the sRI, non-sRI, and normal-RF groups in the training and testing cohorts. Therefore, the model performance of some machine learning algorithms should be compared, and the most appropriate one is selected for study purposes.

There were some limitations in our study. First, the sample size was relatively small. MRI examination is not a routine examination of the patient with renal function impairment. Second, not all patients underwent renal biopsy to identify the degree of renal fibrosis. However, the limited amount of tissue biopsy could not reflect the general pathological changes of DN. Third, we did not perform external validation for the retrospective study. We would like to recruit some patients from other institutions for future external validation.

In conclusion, the T2WI-based model outperformed in assessing the renal function status and the degree of renal fibrosis in DN. The mMRI-TA model based on T2WI and DWI and BOLD measurements can improve the model performance in assessing renal function.

## Data availability statement

The original contributions presented in the study are included in the article/supplementary material. Further inquiries can be directed to the corresponding authors.

## Ethics statement

Studies using patients’ database have had initial ethical approval from the Guangdong General Hospital, Guangdong Academy of Medical Sciences Research Ethics Committee, subject to prior independent scientific review. The Scientific Review Committee (GDREC) approved the study protocol (No. GDREC2017253H). The patients/participants provided their written informed consent to participate in this study.

## Author contributions

WC, LZ, GC, BZ, ZL, YZ, JL, WW, and XM conceived the idea for the study. WC, LZ, and GC conducted the statistical analysis. WC, ZL, YZ, and JL were responsible for the initial draft of the report. WC, GC, JL, WW, and XM contributed to subsequent drafts and all authors were involved in the final draft. WC and XM were responsible for supervision. XM acts as guarantor for the final manuscript. All authors contributed to the article and approved the submitted version.
