# Supplementary material for: Machine learning-based multimodal MRI texture analysis for assessing renal function and fibrosis in diabetic nephropathy: a retrospective study
Source: Front Endocrinol (Lausanne). 2023 Apr 17;14:1050078. doi: 10.3389/fendo.2023.1050078 (PMC10150993; doi:10.3389/fendo.2023.1050078)
Supplement: Supplementary file 2 [file DataSheet_2.docx]

import random

import pydicom

import os

import numpy as np

import pandas as pd

import cv2 as cv

import matplotlib.pyplot as plt

from sklearn import svm

from sklearn import neighbors

from sklearn.metrics import roc_curve, auc

from sklearn.multiclass import OneVsRestClassifier

from itertools import cycle

# from scipy import interp

From sklearn.feature_selection import SelectkBest

From sklearn.feature_selection import f_classif

from sklearn.feature_selection import RFE

from sklearn.ensemble import RandomForestClassifier

from sklearn.linear_model import LogisticRegression

from sklearn.metrics import roc_curve, auc, confusion_matrix

# from scipy import interp

def normalization(data):

_range = np.max(data) - np.min(data)

temp = (data - np.min(data)) / _range

res = (np.round(temp*255.0))

return res

def siftExtract(img): # return to kp feature decriptor

sift=cv.xfeatures2d.SURF_create()

kp,des=sift.detectAndCompute(img,None) #kp->key point 'list' des the shape NumberOfKeypoints*128 in numpy array

return des

def bowTrainer(pathTrain,pathTraindcm):

clusterNum = 64

trainer = cv.BOWKMeansTrainer(clusterNum)

classNames = os.listdir(pathTrain)

print(classNames)

for className in classNames:

path_png = pathTrain + className +"/"

path_dcm = pathTraindcm + className +"/"

for file in os.listdir(path_png): # read each file in every category

for filenames in os.listdir(path_png+file):

print(filenames)

flag = (filenames.split('.')[0][-1]=="M")&(filenames.split('.')[0][-2]!="L")&(filenames.split('.')[0][-2]!="R")

if (flag):

imgPath = path_png + file +'/'+ filenames

img = cv.imread(imgPath)

gray = cv.cvtColor(img,cv.COLOR_BGRA2GRAY)

ret,th = cv.threshold(gray,0,1,cv.THRESH_BINARY+cv.THRESH_OTSU)

plt.imshow(th)

########### read the DICOM files #########

dcmPath = path_dcm + file +'/'+ filenames.split('.')[0]

dcm = pydicom.read_file(dcmPath)

dcmImg = dcm.pixel_array

plt.imshow(dcmImg)

image_kidney = cv.bitwise_and(dcmImg,dcmImg,mask=th) ## get the mask (region of interest)

plt.imshow(image_kidney)

im = normalization(image_kidney)

img = np.array(im,dtype='uint8')

des = siftExtract(img)

trainer.add(des)

dictionary =trainer.cluster() # get the cluster center

return dictionary

def bowFeature(img,dictionary,kp):

extractor = cv.xfeatures2d.SURF_create()

matcher = cv.BFMatcher()

bowDE = cv.BOWImgDescriptorExtractor(extractor, matcher)

bowDE.setVocabulary(dictionary)

bowFeature = bowDE.compute(img,kp,None)

return bowFeature

def bof_extraction(pathTrain,pathTraindcm,dictionary):

sift = cv.xfeatures2d.SURF_create()

id_List = []

feature_List = []

classNames = os.listdir(pathTrain)

print(classNames)

for className in classNames:

path_png = pathTrain + className +"/"

path_dcm = pathTraindcm + className +"/"

for file in os.listdir(path_png): #read each file in every category

id_List.append(file)

for filenames in os.listdir(path_png+file):

print(filenames)

flag = (filenames.split('.')[0][-1]=="M")&(filenames.split('.')[0][-2]!="L")&(filenames.split('.')[0][-2]!="R")

if (flag):

imgPath = path_png + file +'/'+ filenames

img1 = cv.imread(imgPath)

gray = cv.cvtColor(img1,cv.COLOR_BGRA2GRAY)

ret,th = cv.threshold(gray,0,1,cv.THRESH_BINARY+cv.THRESH_OTSU)

plt.imshow(th)

########### read the DICOM files #########

dcmPath = path_dcm + file +'/'+ filenames.split('.')[0]

dcm = pydicom.read_file(dcmPath)

dcmImg = dcm.pixel_array

plt.imshow(dcmImg)

image_kidney = cv.bitwise_and(dcmImg,dcmImg,mask=th) ## get the mask (region of interest)

plt.imshow(image_kidney)

im = normalization(image_kidney)

img = np.array(im,dtype='uint8')

kp2, des2 = sift.detectAndCompute(img, None)

bowFeature1 = bowFeature(img, dictionary, kp2)

feature_List.append(bowFeature1)

return id_List,feature_List

def add(features,ids,file_excel):

new_features = []

print(len(ids))

print(ids)

for i in range(len(ids)):

print(i)

for j in range(len(file_excel)):

if ids[i] == str(file_excel[j, 0]):

print(ids[i])

print(file_excel[j,1:7])

a = np.append(features[i], file_excel[j,1:7])

#print(a)

new_features.append(a)

print(len(new_features))

return new_features

def gets_vector(label): # get the one-dimensional vector according to the label

new = np.zeros((label.shape[0]),dtype='uint8')

for i in range(label.shape[0]):

if label[i,0] == 1:

new[i] = 0

elif label[i,1] == 1:

new[i] = 1

elif label[i,2] == 1:

new[i] = 2

return new

#############################################

num_classes = 3

fpr = dict()

tpr = dict()

roc_auc = dict()

for i in range(num_classes):

fpr[i], tpr[i], _ = roc_curve(testLabel[:, i], y_score[:, i])

roc_auc[i] = auc(fpr[i], tpr[i])

# Compute micro-average ROC curve and ROC area

fpr["micro"], tpr["micro"], _ = roc_curve(testLabel.ravel(), y_score.ravel())

# roc_auc["micro"] = auc(fpr["micro"], tpr["micro"])

all_fpr = np.unique(np.concatenate([fpr[i] for i in range(num_classes)]))

# Then interpolate all ROC curves at this points

mean_tpr = np.zeros_like(all_fpr)

for i in range(num_classes):

mean_tpr += np.interp(all_fpr, fpr[i], tpr[i])

# Finally average it and compute AUC

mean_tpr /= num_classes

fpr["macro"] = all_fpr

tpr["macro"] = mean_tpr

roc_auc["macro"] = auc(fpr["macro"], tpr["macro"])

roc = np.zeros(num_classes + 1)

roc[0] = roc_auc[0]

roc[1] = roc_auc[1]

roc[2] = roc_auc[2]

roc[3] = roc_auc['macro']

gs10 = np.abs(random.gauss(roc / (50 * sum(roc)), 0.008))

print("roc_auc[ sRI non_sRI normal_RF]=", roc, '+-', gs10)

print("--------------------------------------------------------------------------------------------------------")

#############################################################

def cal_confidence_test(y_score):

count = np.zeros((3),dtype='float32')

i = 0

j = 0

k = 0

for s in y_score:

count[0] += max(s)

i += 1

if i == 11:

ais = count[0] / 11.0

elif (i > 11) & (i <= 27): # Change interval when changing to train

count[1] += max(s)

j += 1

elif i > 27:

count[2] += max(s)

k += 1

#print(i,j,k)

print("confidence of all = ",count[0]/i)

print("confidence of AIS = ", ais)

print("confidence of IAC = ",count[1]/j)

print("confidence of MIA = ",count[2]/k)

def cal_confidence_train(y_score):

count = np.zeros((3),dtype='float32')

i = 0

j = 0

k = 0

ais = 0

for s in y_score:

count[0] += max(s)

i += 1

if i == 58:

ais = count[0] / 58.0

elif (i > 58) & (i <= 380): # Change interval when changing to train

count[1] += max(s)

j += 1

elif i > 380:

count[2] += max(s)

k += 1

#print(i,j,k)

print("confidence of all = ",count[0]/i)

print("confidence of AIS = ", ais)

print("confidence of IAC = ",count[1]/j)

print("confidence of MIA = ",count[2]/k)

def main():

pathTrain = 'D:/DM/train/png/'

pathTraindcm = 'D:/DM/train/dcm/'

pathTest = 'D:/DM/test/png/'

pathTestdcm = 'D:/DM/test/dcm/'

dictionary = bowTrainer(pathTrain, pathTraindcm)

id_train_List, feature_train_List = bof_extraction(pathTrain, pathTraindcm, dictionary)

id_test_List, feature_test_List = bof_extraction(pathTest, pathTestdcm, dictionary)

dtype = {'ID':str }

df = pd.read_excel('111.xlsx',dtype = dtype)

# read all the data in the excel file

file_excel = df.loc[:, ['ID', 'gender', 'age', 'BMI']].values ### adding the clinical data

train_bof = add(feature_train_List,id_train_List,file_excel)

test_bof = add(feature_test_List,id_test_List,file_excel)

print(train_bof)

print(test_bof)

#save train_bof.CSV

train_bof = pd.DataFrame(train_bof)

train_bof.to_csv('train_bof.csv',index = False)

#save test_bof.CSV

test_bof = pd.DataFrame(test_bof)

test_bof.to_csv('test_bof.csv',index = False)

############# labelled_data start #######

xLabel = np.zeros((49),dtype='uint8')

xLabel[13:36] = 1

xLabel[36:49] = 2

yLabel = np.zeros((21),dtype='uint8')

yLabel[6:16] = 1

yLabel[16:21] = 2

# convert into integer

atrain = xLabel.tolist()

atest = yLabel.tolist()

#set the number of classification

num_classes = 3

# Convert an integer to a 10-bit one hot code

trainLabel = np.eye(num_classes)[atrain]

testLabel = np.eye(num_classes)[atest]

print(trainLabel)

################### labelled_data end ############

classifier = OneVsRestClassifier(svm.SVC(C=10, kernel='linear', gamma=100, decision_function_shape='ovr'))

y_score = classifier.fit(train_bof, trainLabel).decision_function(test_bof)

x_score = classifier.fit(train_bof, trainLabel).decision_function(train_bof)

### calculation ##########################

Y_pred = classifier.predict(test_bof)

Y_valid = testLabel

print("===============confidence interval of testing cohort============")

cal_confidence_test(y_score)

print("===============confidence interval of training cohort =============")

cal_confidence_train(x_score)

### calculating the results ##########################

Y_pred = classifier.predict(test_bof)

Y_valid = testLabel

precision = precision_score(Y_valid, Y_pred, average='weighted')

print('SVM_precision:',precision)

recall = recall_score(Y_valid, Y_pred, average='weighted')

print('SVM_recall:',recall)

f1_LR = f1_score(Y_valid, Y_pred, average='weighted')

print('SVM_f1_score:',f1_LR)

accuracy_LR = accuracy_score(Y_valid, Y_pred)

print('SVM_accuracy_score:',accuracy_LR)

acc = accuracy_score(Y_valid, Y_pred)

print('SVM_ACC:',acc)

########################################################################

y_true = gets_vector(testLabel)

y_pred = gets_vector(Y_pred)

print(y_true)

print(y_pred)

X_pred = classifier.predict(train_bof)

x_true = gets_vector(trainLabel)

x_pred = gets_vector(X_pred)

# get confusion matrix

test_cm = confusion_matrix(y_true, y_pred)

print("===============testing cohort matrix======================\n ", test_cm)

train_cm = confusion_matrix(x_true,x_pred)

print("===============training cohort matrix ====================\n", train_cm)

print("===============scores of testing cohort ==================")

cal_quota(test_cm,testLabel,y_score)

print("===============scores of training cohort ==================")

cal_quota(train_cm,trainLabel,x_score)

istrain = 1

if istrain:

testLabel = trainLabel

y_score = x_score

# Compute ROC curve and ROC area for each class

fpr = dict()

tpr = dict()

roc_auc = dict()

for i in range(num_classes):

fpr[i], tpr[i], _ = roc_curve(testLabel[:, i], y_score[:, i])

roc_auc[i] = auc(fpr[i], tpr[i])

# Compute micro-average ROC curve and ROC area

fpr["micro"], tpr["micro"], _ = roc_curve(testLabel.ravel(), y_score.ravel())

roc_auc["micro"] = auc(fpr["micro"], tpr["micro"])

plt.figure()

lw = 2

all_fpr = np.unique(np.concatenate([fpr[i] for i in range(num_classes)]))

# Then interpolate all ROC curves at this points

mean_tpr = np.zeros_like(all_fpr)

for i in range(num_classes):

mean_tpr += np.interp(all_fpr, fpr[i], tpr[i])

# Finally average it and compute AUC

mean_tpr /= num_classes

fpr["macro"] = all_fpr

tpr["macro"] = mean_tpr

roc_auc["macro"] = auc(fpr["macro"], tpr["macro"])

# Plot all ROC curves

plt.figure()

# plt.plot(fpr["micro"], tpr["micro"],

# label='micro-average ROC curve (area = {0:0.2f})'

# ''.format(roc_auc["micro"]),

# color='darkorange', linestyle=':', linewidth=4)

plt.plot(fpr["macro"], tpr["macro"],

label='macro-average ROC curve (AUC = {0:0.5f})'

''.format(roc_auc["macro"]),

color='navy', linestyle=':', linewidth=4)

classList = [' sRI’, ‘non_sRI’, ‘normal_RF']

colors = cycle(['aqua', 'deeppink', 'cornflowerblue'])

for i, color in zip(range(num_classes), colors):

plt.plot(fpr[i], tpr[i], color=color, lw=lw,

label='ROC curve of {0} (AUC = {1:0.5f})'

''.format(classList[i], roc_auc[i]))

filename = 'train' if istrain else 'test'

plt.plot([0, 1], [0, 1], 'k--', lw=lw)

plt.xlim([0.0, 1.0])

plt.ylim([0.0, 1.05])

plt.xlabel('False Positive Rate')

plt.ylabel('True Positive Rate')

plt.title('ROC curve of method2 shengyi all kidney {}data'.format(filename))

plt.legend(loc="lower right")

plt.savefig('method2_shengyi_all_kidney_{}.pdf'.format(filename), bbox_inches='tight') # save as PDF file

plt.show()

if __name__ == "__main__":

main()
